# Therapeutic potential of transcutaneous auricular vagus nerve stimulation in cognitive impairment: insights from preclinical and clinical studies

**DOI:** 10.3389/fneur.2026.1749948

**Published:** 2026-04-07

**Authors:** Di Pan, Jifei Sun, Wenchao Mao, Huaxin Shi

**Affiliations:** 1 Beijing Genertec Aerospace Hospital, Beijing, China; 2 Shunyi Hospital, Beijing Hospital of Traditional Chinese Medicine, Beijing, China; 3 Guang’anmen Hospital, China Academy of Chinese Medical Sciences, Beijing, China; 4 China Academy of Chinese Medical Sciences, Beijing, China

**Keywords:** cognitive impairment, mild cognitive impairment, neuromodulation, post-stroke cognitive impairment, transcutaneous auricular vagus nerve stimulation

## Abstract

Transcutaneous auricular vagus nerve stimulation (taVNS) is being investigated as a non-invasive neuromodulatory approach for cognitive impairment (CI). This review evaluates the existing preclinical and clinical evidence regarding its potential efficacy and mechanisms of action in conditions such as mild cognitive impairment, post-stroke cognitive impairment, and other forms of CI. Preclinical models suggest that taVNS may influence multiple pathways, including neurotransmitter regulation, neuroinflammation, apoptosis, and synaptic plasticity. Clinically, some small-scale studies report modest improvements in cognitive metrics, but the evidence remains preliminary due to methodological limitations such as small sample sizes, heterogeneous parameters, and short intervention durations. Significant challenges, including the lack of standardized protocols, inadequate sham controls, and an underdeveloped mechanistic understanding, currently hinder the interpretation and translation of findings. Future research necessitates large-scale, rigorously controlled trials and deeper mechanistic studies to determine whether taVNS has a definitive role in the clinical management of cognitive impairment.

## 1 Introduction

Cognitive impairment (CI) is a common clinical condition characterized by significant decline in one or more cognitive domains, including memory, language, attention, executive function, and orientation (1). This impairment is a core clinical feature of various diseases. In neurodegenerative disorders such as Alzheimer’s disease (AD), CI often serves as a primary symptom and tends to progressively worsen (2). Following cerebrovascular events, it is also a common and severely debilitating complication known as post-stroke cognitive impairment (PSCI) (3). Furthermore, CI is prevalent among patients with psychiatric disorders and is closely associated with various chronic systemic diseases (4). Even during physiological aging, many middle-aged and elderly individuals experience subjective cognitive decline or mild objective cognitive deterioration, which may serve as precursors to more severe CI.

From an epidemiological perspective, the disease burden caused by CI is extremely heavy and continues to intensify with the global aging population (5). Its prevalence increases significantly with age: among those aged 65 and older, the prevalence of dementia is approximately 5–10%, rising to 20–30% or higher in individuals aged 80 and above (6). CI and related dementias not only severely impact patients’ ability to live independently and their quality of life but also impose heavy psychological, physical, and financial burdens on caregivers (7). Taking AD as an example, the global patient population has reached tens of millions, with incidence rates continuing to rise (8). PSCI also occurs in 20–50% of stroke patients (9). Given the high prevalence and widespread nature of CI across diseases, existing pharmacological treatments remain limited in efficacy and associated with significant adverse effects (10). Consequently, developing novel, safe, and effective intervention strategies has become an urgent priority in neuroscience and clinical medicine.

The pathogenesis of CI exhibits high complexity, involving the interplay of multi-system, multi-level pathophysiological processes (11). Its core mechanisms can be summarized into the following four interrelated aspects: (1) Progressive damage to neural structures and functions, primarily manifested as pathological accumulation of abnormal proteins, neuronal degeneration and death, loss of synaptic connections, and impaired cerebral vascular integrity (12, 13); (2) Cerebrovascular dysfunction, where cerebral atherosclerosis, localized infarcts, or microbleeds cause insufficient cerebral perfusion and hypoxic–ischemic injury, subsequently damaging critical brain regions such as the hippocampus and prefrontal cortex that are closely associated with learning and memory (9); (3) Neurochemical imbalance, most notably characterized by significant acetylcholine deficiency within the cholinergic system, alongside dysfunction in glutamatergic excitotoxicity and neurotransmitter systems involving dopamine and serotonin, collectively impairing efficient neural signaling and integration (14, 15); (4) The cumulative effects of systemic risk factors, including age-related declines in cellular repair capacity, carriage of genetic susceptibility genes, persistent neuroinflammatory responses, and oxidative stress-induced damage to biomolecules. These factors mutually exacerbate each other, collectively forming the deep biological basis for progressive cognitive decline (16–18).

Currently, drug intervention remains the primary treatment approach for CI. Among pharmacological therapies, symptom-improving medications such as donepezil and causative-targeted drugs like lecanemab—which has garnered significant attention in recent years—constitute the current treatment regimen (19). However, the former typically only alleviate symptoms without halting disease progression and are often accompanied by certain side effects (20). The latter face core limitations including high cost, potential safety risks such as cerebral edema, and a narrow therapeutic window—being applicable only to early-stage patients (21). Compounding this, clinical diagnosis is often delayed, meaning most patients are diagnosed after missing the optimal intervention window (22). Overall, existing medications are largely confined to symptom management, with significant interindividual variability in efficacy (23). Therefore, exploring safe, effective, and accessible non-pharmacological interventions holds substantial clinical significance and practical value for improving cognitive and emotional functioning.

## 2 From concept to intervention: the realization of transcutaneous auricular vagus nerve stimulation

**Figure 1:**
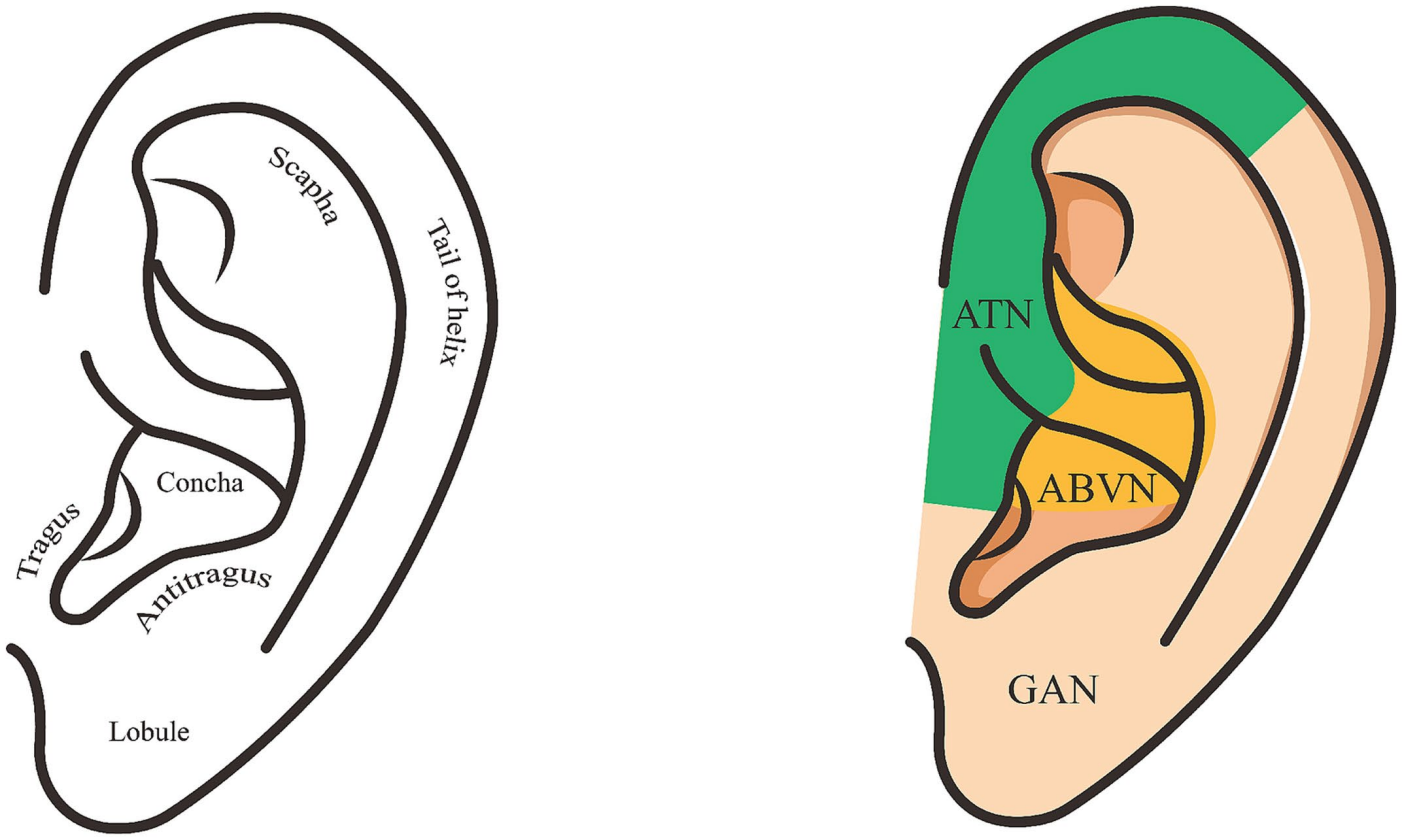
The figure illustrates the distribution of the vagus nerve in the ear. The left diagram shows the names of various parts of the ear; the yellow areas in the right diagram indicate the distribution of the vagus nerve.

**Figure 2:**
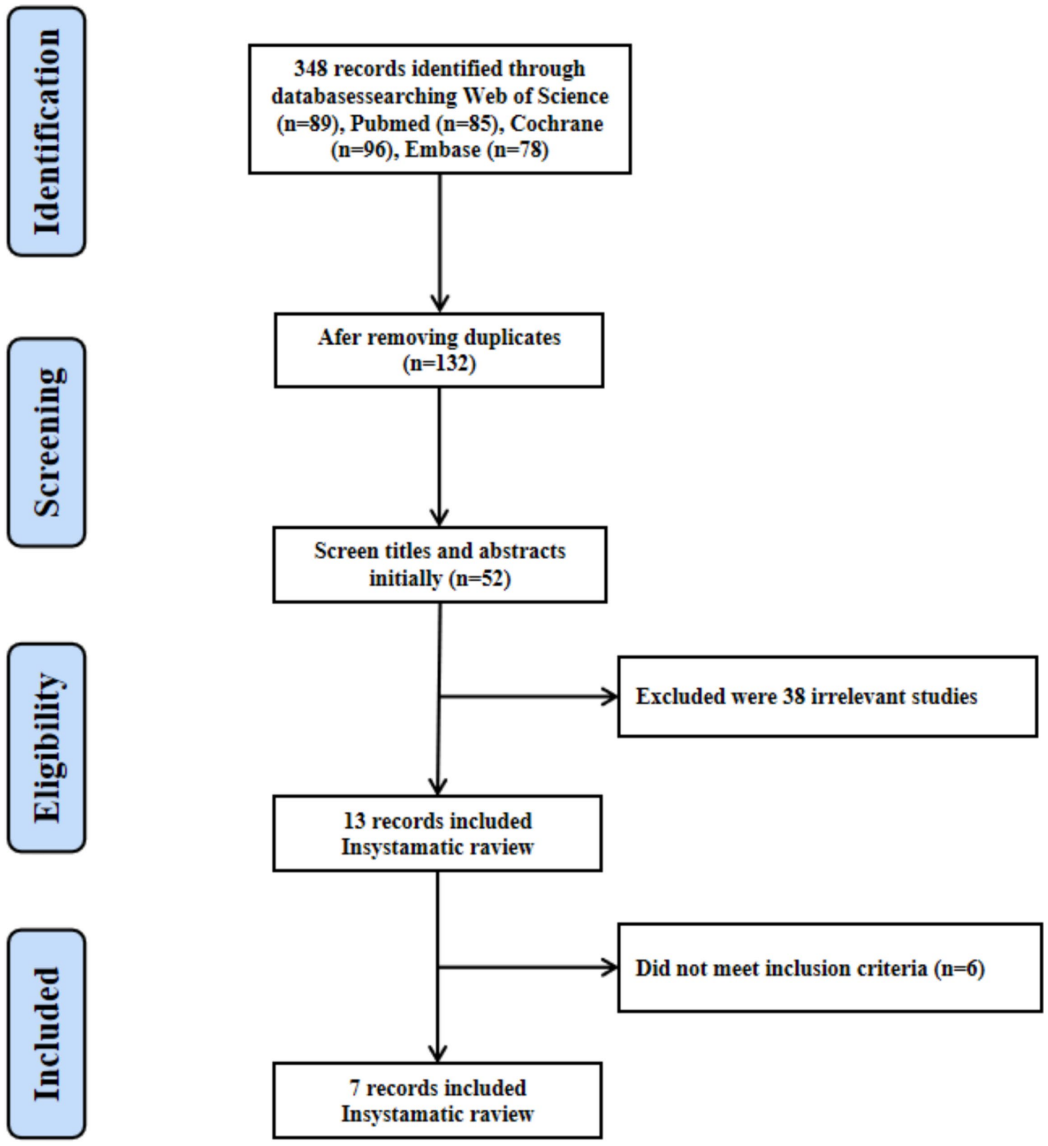
Flow chart of study selection.

The vagus nerve, as the tenth cranial nerve, is the longest and most widely distributed mixed nerve in the human body, characterized by complex anatomy and diverse functions (24). It originates from multiple nuclei within the medulla oblongata, including the ambiguus nucleus, dorsal vagal nucleus, solitary tract nucleus, and spinal trigeminal nucleus. Its fiber composition encompasses four distinct types: special visceral motor, general visceral motor, general visceral sensation, and general somatic sensation. After exiting the cranium through the foramen magnum, it descends within the carotid sheath, with its branches extensively innervating organs in multiple regions including the head, neck, chest, and abdomen (25, 26). The main branches include: (1) the auricular branch, which innervates the skin of the external auditory canal (Figure 1); (2) the pharyngeal branch and superior laryngeal nerve/recurrent laryngeal nerve, which innervate the muscles of the pharynx and larynx; (3) the visceral branches, which extend into the thoracic and abdominal cavities to regulate cardiac, pulmonary, esophageal, and gastrointestinal functions. Functionally, approximately 80% of vagus nerve fibers are afferent, responsible for transmitting diverse sensory information (27, 28). Among these, common somatic afferent fibers project sensory information from the external auditory canal to the trigeminal spinal nucleus, while common visceral afferent fibers collect information from thoracic and abdominal organs and the aortic body, projecting to the caudate nucleus of the solitary tract. Additionally, special visceral afferent fibers originating from the epiglottis are responsible for taste transmission, with their information ultimately projecting to the nucleus tract solitary (NTS) (29). Crucially, the NTS serves as the primary afferent relay station for the vagus nerve. Beyond receiving these signals, it further projects to brainstem nuclei such as the locus coeruleus and parabrachial nucleus. Through extensive connections with higher centers including the thalamus, amygdala, hippocampus, and prefrontal cortex, it participates in regulating diverse physiological and pathophysiological processes, including visceral reflexes, inflammatory responses, emotional cognition, cardiovascular homeostasis, and seizure control (30, 31).

Since receiving United States Drug and Food Administration approval in 2005 for treatment-resistant depression, vagus nerve stimulation (VNS) has gradually expanded its indications into the field of CI (32). Accumulating evidence suggests that VNS significantly improves vascular cognitive impairment (33), traumatic brain injury-related cognitive deficits (34), and even AD (35). Despite its promising prospects, the surgical risks, potential infections, and high costs associated with traditional invasive implant-based VNS constitute major barriers to its clinical adoption, driving an urgent need for non-invasive VNS technologies (36).

Based on the unique anatomical feature of the auricular vagus nerve as the sole superficial vagus nerve branch in the human body (37), transcutaneous auricular vagus nerve stimulation (taVNS) serves as an innovative non-invasive neuromodulation technique. By precisely stimulating the auricular vagus nerve branch, it delivers therapeutic effects while successfully overcoming the invasive limitations of traditional VNS. The development of taVNS integrates the wisdom of Eastern and Western medical philosophies. Its conceptual foundation traces back to traditional Chinese medicine’s auricular therapy, practiced for millennia (38, 39). Traditional Chinese medical theory views the auricle as a holographic projection of an “inverted fetus,” connected to all internal organs via meridians. Stimulating specific auricular points is believed to regulate corresponding organ functions (40). This understanding provided the initial theoretical and practical basis for auricular therapy. By the 20th century, modern neuroanatomy achieved a pivotal breakthrough: research confirmed dense innervation of the concha and antrum regions of the human auricle by the auricular branch of the vagus nerve (41). This discovery transformed the traditional “meridian” connection into a defined “nerve” pathway, providing scientific justification for activating the vagus nerve through auricular stimulation. Entering the 21st century, taVNS transitioned from theory to practice. Around 2000, researchers formally introduced the concept of “transcutaneous auricular vagus nerve stimulation,” systematically applying transcutaneous electrical nerve stimulation devices to deliver precise electrical stimulation to the concha region as an alternative to implantable cervical stimulation (42). Subsequent studies using functional magnetic resonance imaging (fMRI) and other techniques confirmed that taVNS effectively activates brainstem nuclei such as the solitary tract nucleus and higher-order brain regions, regulating the autonomic nervous system and inflammatory responses (43–45). As its mechanisms became progressively elucidated, clinical applications expanded from initial indications like epilepsy and depression to encompass migraine, CI, and systemic lupus erythematosus (18, 45–47).

Existing clinical studies indicate that taVNS significantly improves symptoms such as CI, demonstrating efficacy comparable to VNS (48). It offers advantages including non-invasiveness, safety, and ease of operation, showcasing broad clinical potential. However, current evidence primarily stems from small-sample, short-term trials, with a lack of high-quality research systematically validating the long-term efficacy and neural mechanisms of taVNS for treating CI (49, 50). This review aims to synthesize existing clinical and experimental evidence to comprehensively evaluate the efficacy of taVNS in treating cognitive impairment, explore its mechanisms of action, and provide theoretical foundations and practical guidance for advancing the clinical translation of this technology. Specifically, given that current clinical trials primarily focus on functional outcomes and often lack direct evidence of neural changes, we will also review pertinent preclinical animal literature in Section 5. Specifically, while cognitive impairment is also a prevalent feature in psychiatric disorders such as depression, this review primarily focuses on cognitive decline stemming from neurodegenerative, vascular, and systemic organic pathologies. In these contexts, cognitive dysfunction serves as a primary target for intervention rather than a secondary outcome of mood dysregulation.

## 3 Methods

### 3.1 Search strategy

A systematic literature search was conducted across four major electronic databases—PubMed, Embase, Web of Science, and Cochrane Library—from their inception until November 10, 2025. The aim was to identify clinical studies investigating the use of taVNS in populations with CI. The search strategy combined Medical Subject Headings (MeSH) with free-text keywords, focusing on terms and their variations such as “transcutaneous auricular vagus nerve stimulation,” “taVNS,” “transcutaneous vagus nerve stimulation,” “tVNS,” “auricular transcutaneous vagus nerve stimulation,” “atVNS,” “cognitive impairment,” “mild cognitive impairment,” “post-stroke cognitive impairment,” “vascular cognitive impairment,” “Alzheimer’s disease,” “dementia.” Study types included randomized controlled trials (RCTs), single-arm trials, case reports, and non-randomized controlled trials.

### 3.2 Research screening process

The screening process was conducted in two phases. First, three reviewers (DP, JS, and WM) independently screened titles and abstracts of all retrieved records. If eligibility could not be determined from the title and abstract alone, the full text was obtained for further assessment. Discrepancies were resolved through discussion or by consulting a fourth reviewer (HS). To improve efficiency and accuracy, retrieved records were imported into EndNote software (Analytics Inc., Philadelphia, United States) for deduplication and initial screening. Eligible records were then transferred to Zotero 5.0 (Digital Scholar LLC, Vienna, United States) for full-text review and final inclusion confirmation. No restrictions were placed on publication date or language.

Inclusion criteria were as follows: (1) Original clinical research articles; (2) Studies involving participants with cognitive impairment associated with neurological, vascular, or systemic organic pathologies; (3) Intervention involving taVNS or transcutaneous vagus nerve stimulation (tVNS) applied to the auricular region; (4) Study designs including RCTs, single-arm trials, case reports, or non-randomized controlled trials; (5) Articles published in English.

Exclusion criteria included: (1) Non-original research (e.g., reviews, meta-analyses, editorials, conference abstracts); (2) Studies using non-electrical auricular interventions; (3) Studies utilizing transcutaneous cervical vagus nerve stimulation (tcVNS) or other non-auricular stimulation sites, to maintain anatomical homogeneity; (4) Lack of relevant cognitive or safety outcome data; (5) Inaccessible data after contacting authors; (6) Studies not focused on cognitive impairment; (7) Studies focusing primarily on CI secondary to psychiatric disorders (e.g., depression, schizophrenia) to maintain pathophysiological homogeneity.

### 3.3 Data extraction

Data extraction and quality assessment were performed independently by two reviewers (JS and DP). For studies with missing or unclear information, corresponding authors were contacted via email to obtain additional details. Extracted data included: (1) First author and publication year; (2) Study population characteristics; (3) Stimulation site; (4) Intervention protocol details; (5) Primary cognitive outcome measures. Extracted data were summarized in tabular form (Table 1) to facilitate comparison and synthesis (Figure 2).

**Table 1:** Applications of taVNS in clinical cognitive disorders.

References	Characteristics (n)	Study design	treatment	Treatment duration	Main site	Stimulation parameters	Assessment Tools (Objective/Subjective)	Key Outcomes (Pre vs. Post / Score Change)	Conclusions
Wang et al. (48)	MCI (60)	RCT (Sham-controlled)	taVNS vs. Sham	24 weeks	Concha (taVNS) vs. scaphoid (sham)	Mixed frequencies: 20 Hz for 10s & 100 Hz for 50s; Intensity: 0.6–1.0 mA	Obj: MoCA-B, AVLT-H Subj: PSQI	MoCA-B: Significant improvement in taVNS group vs. Sham (*p* < 0.05), AVLT-H: N5 and N7 scores significantly increased (*p* < 0.001)	1. Significantly improved global cognition and memory in MCI 2. Intervention was safe with one minor adverse event; 3. Represents an effective non-pharmacological option for MCI
Murphy et al. (63)	MCI (50)	RCT (Sham-controlled)	tVNS vs. Sham	Acute (During MRI scan)	Active: Left tragus; Sham: Left ear lobe	Frequency: 20 Hz; Pulse width: 50 μs; Intensity: set at 80% of individual discomfort threshold (Mean: 7.3 mA)	Obj: Resting-state fMRI	fMRI: Altered functional connectivity in semantic and salience networks (temporal/parietal lobes) and from hippocampi to cortical/subcortical clusters vs. sham.	1. tVNS modifies activity in brain networks associated with AD. 2. Provides evidence of afferent target engagement in MCI patients.
Chen et al. (52)	PSCI (1)	Single-arm pilot	taVNS	8 weeks	Right ear	Mixed frequencies: 20 Hz for 7 s / 4 Hz for 3 s	Obj: MoCA, STT, DTI	MoCA: Score improved from 7 (baseline) to 11 (post-treatment), STT: Completion time decreased significantly	1. Feasible and potentially improves global cognition and executive function in long-term PSCI 2. Induced white matter remodeling 3. Mood and sleep improvements observed
Wang et al. (54)	CKD related cognitive impairment (36)	RCT (Sham-controlled,)	taVNS vs. Sham	2 weeks	Left cymba conchae	25 Hz; Pulse width: 200 μs; Intensity: max 10 mA	Obj: MoCA, Subj: VAS-F	MoCA: taVNS prevented the intradialytic decline seen in Sham (*p* < 0.001), VAS-F: Fatigue significantly reduced (*p* = 0.004)	Improved cognitive performance and reduced fatigue 2. Alleviated intradialytic decline in cerebral oxygenation 3. Improvement in cerebral rSO₂ partially mediated cognitive benefits 4. Promising non-pharmacological intervention for MHD patients
Dolphin et al. (62)	Amnestic MCI (40)	RCT (Sham-controlled, crossover)	tVNS vs. Sham vs. Baseline	Acute (Single 60-min session)	Auricular branch	8 Hz; Individual amplitude (mean 2.5 mA)	Obj: FNAT, Spatial navigation task, Hemodynamics	FNAT: Recall accuracy significantly improved vs. baseline/sham (*p* = 0.01). Spatial navigation: Time significantly reduced vs. baseline/sham.	1. tVNS is safe and tolerable in aMCI. 2. Acute tVNS improves spatial and associative memory.
Guo et al. (51)	MCI (1)	Case report	taVNS	24 weeks	Bilateral concha	Sparse/dense wave (20/100 Hz); Intensity: 3–8 mA	Obj: MoCA-B, AVLT, fMRI	MoCA-B: Score improved from 19 to 24, AVLT: Scores improved (e.g., N5 from 4 to 3, N7 from 10 to 19)	1. Improved cognitive function and depressive symptoms 2. Increased neural activity in right temporal pole and left medial orbitofrontal gyrus 3. Benefits persisted for 24 weeks post-treatment
Li et al. (53)	PSCI (30)	Single-arm pilot	taVNS + TOT	3 weeks	Left cymba conchae	25 Hz, 30s on/30s off Pulse width: 500 μs; Intensity: 1–10 mA	Obj: MoCA, FMA-UE fNIRS	MoCA: Significant improvement compared to baseline and Sham (*p* < 0.05), FMA-UE: Upper limb function improved (*p* < 0.05)	1. Superior to sham + TOT in improving upper limb function and cognition 2. Enhanced autonomic homeostasis and corticospinal excitability 3. fNIRS revealed task-specific cortical activation 4. Promotes recovery via multi-pathway neuroregulation

Transcutaneous Auricular Vagus Nerve Stimulation, taVNS; Task-Oriented Training, TOT; Chronic Kidney Disease, CKD; Post-Stroke Cognitive Impairment, PSCI; Mild Cognitive Impairment, MCI; Functional Near-Infrared Spectroscopy, fNIRS; Regional Oxygen Saturation, rSO₂; Maintenance Hemodialysis, MHD; MoCA-B, Montreal Cognitive Assessment-Basic; AVLT-H, Auditory Verbal Learning Test; PSQI, Pittsburgh Sleep Quality Index; STT, Shape Trails Test; DTI, Diffusion Tensor Imaging; RAVLT, Rey Auditory Verbal Learning Test; VAS-F, Visual Analog Scale of Fatigue; fMRI, Functional Magnetic Resonance Imaging; FMA-UE, Fugl-Meyer Assessme; Obj, Objective; Subj, Subjective.

## 4 Applications of taVNS in clinical cognitive impairment

**Figure 3:**
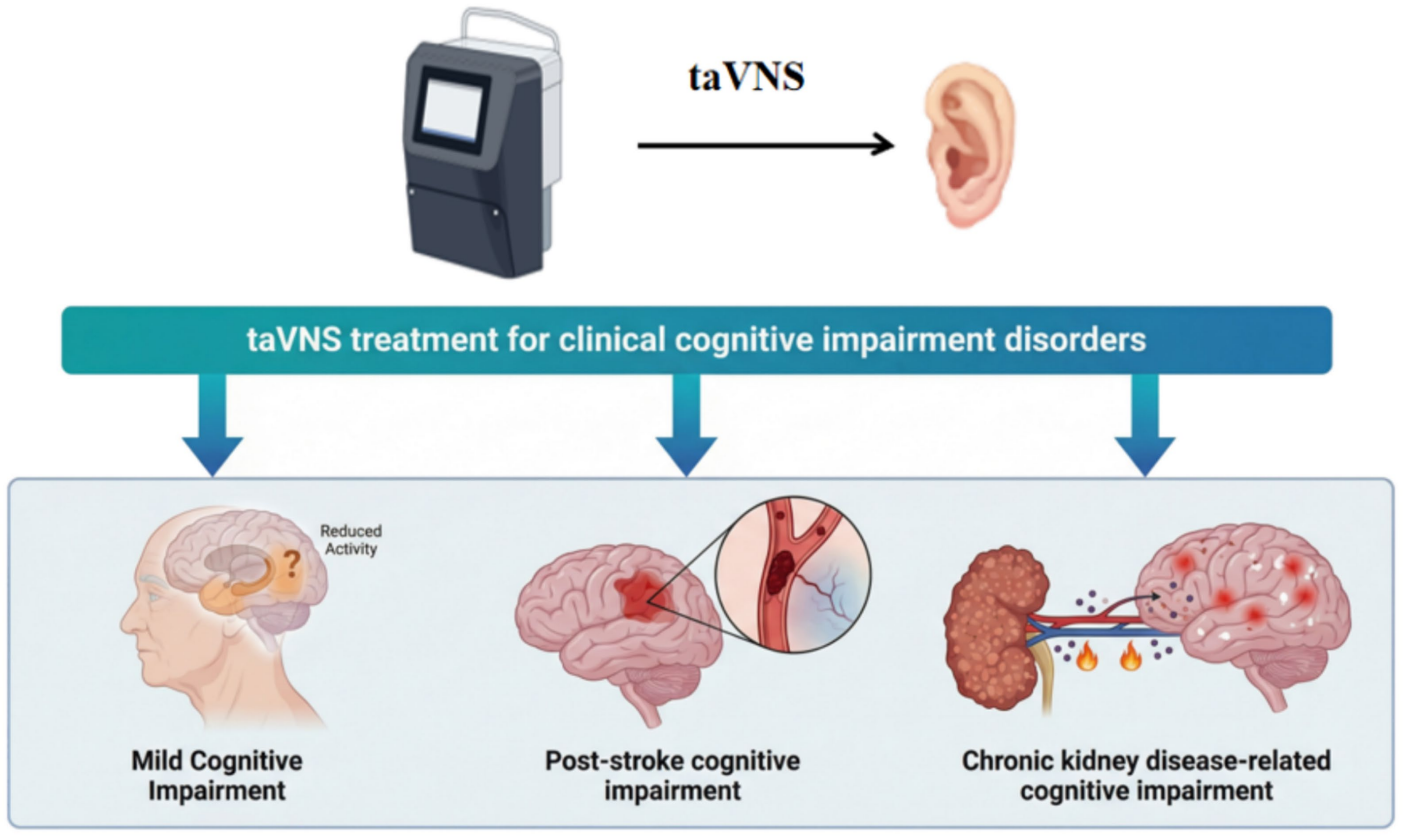
This figure summarizes the application of taVNS in clinical cognitive impairment.

In recent years, taVNS has gained prominence in the treatment of CI. Table 1 summarizes the characteristics and key findings of the included studies. We explicitly categorized outcome measures into objective cognitive tasks and subjective patient-reported outcomes to provide a granular view of efficacy. Quantitative changes in scores and statistical significance are detailed in the table. Clinical studies indicate that taVNS demonstrates therapeutic efficacy across multiple types of CI, including mild cognitive impairment (MCI) (48, 51), post-stroke cognitive impairment (PSCI) (52, 53), and chronic kidney disease (CKD)-related cognitive impairment (54). Although most current research focuses on taVNS’s improvement of mild cognitive impairment, studies on other types of CI remain relatively scarce (Table 1; Figure 3). Although current research predominantly focuses on MCI, emerging preclinical and clinical investigations suggest that taVNS may also hold potential in dementia-related contexts, such as vascular cognitive impairment and dementia (55, 56). However, robust clinical evidence specifically targeting Alzheimer’s disease or other major dementia subtypes remains limited, and further high-quality trials are needed to establish its efficacy and mechanisms in these populations (Table 1; Figure 3).

### 4.1 Application of taVNS in mild cognitive impairment

MCI refers to a clinical state where patients exhibit impairment in memory or other cognitive functions, yet their ability to perform daily activities remains largely unaffected, falling short of the severity of dementia. It is considered a transitional phase between normal aging and dementia (57). Among individuals aged 65 and older, the annual incidence of MCI ranges from approximately 5 to 10%. Annually, about 10–15% of these patients progress to dementia, a rate significantly higher than the 1–2% observed in the general elderly population (58, 59). Therefore, the MCI stage, particularly its early phase, is recognized as a “golden window” for intervention. By controlling modifiable risk factors and adopting healthy lifestyles, it is possible to delay or even prevent the progression of some MCI cases to dementia (60).

A double-blind randomized controlled trial by Wang et al. systematically evaluated the efficacy and safety of taVNS in MCI patients. Sixty MCI patients were randomly assigned to either the taVNS group or a sham stimulation group for 24 weeks of home-based self-administration (61). Results demonstrated significant improvements in overall cognitive function and memory in the taVNS group compared to sham stimulation, with good safety profiles. Only one participant with a history of tympanic membrane perforation reported mild, reversible discomfort. This study suggests taVNS, as an effective, safe, and easily scalable non-pharmacological therapy, may help delay the progression of MCI to dementia. Guo et al. explored the efficacy and neural mechanisms of taVNS in patients with MCI comorbid with depression through a case study (51). A 71-year-old female patient underwent 24 weeks of taVNS intervention combined with functional magnetic resonance imaging (fMRI) assessment. Post-treatment, the patient demonstrated significant improvements in both cognitive function and depressive symptoms. fMRI further revealed enhanced functional activity in brain regions associated with cognition and emotion, including the right temporal pole and left medial orbitofrontal cortex. Symptoms remained remitted during follow-up, indicating that taVNS holds potential therapeutic value for MCI patients with depressive symptoms. This provides preliminary clinical and neuroimaging evidence supporting the application of this therapy in managing cognitive-emotional comorbidity. In addition to long-term interventions, recent studies using the broader terminology of tVNS have highlighted the acute benefits of auricular stimulation. For instance, single-session tVNS has been shown to rapidly improve spatial and associative memory in patients with amnestic MCI (62), while concurrent functional neuroimaging reveals that it immediately modulates functional connectivity within semantic and hippocampal networks, providing direct evidence of afferent target engagement (63).

### 4.2 Application of taVNS in post-stroke cognitive impairment

PSCI is one of the most common complications following stroke (3). Clinical observations indicate that over one-third of patient’s exhibit significant cognitive impairment in the early post-stroke period, and the severity of post-stroke cognitive impairment is closely associated with patient prognosis (64). Compared to non-stroke individuals and stroke survivors without cognitive impairment, patients with PSCI experience accelerated cognitive decline (65). Therefore, early identification of its severity and implementation of targeted interventions can significantly improve overall patient outcomes.

Wang et al. conducted an 8-week home-based taVNS intervention on a 71-year-old male patient with persistent PSCI 2.5 years after stroke onset (52). The patient was assessed at four time points using cognitive scales and diffusion tensor imaging (DTI). Results showed that after 8 weeks of taVNS intervention, the patient’s Montreal Cognitive Assessment score improved, and the completion time for the Shape Trajectory Test-B was significantly reduced. DTI further revealed improved white matter integrity in the dorsolateral prefrontal cortex (DLPFC), a region closely associated with cognitive function, along with an increase in the number of white matter fiber tracts connecting bilateral DLPFC. This study demonstrates that taVNS not only helps improve cognitive function in long-term PSCI patients but also promotes white matter structural remodeling, offering a promising new strategy for cognitive rehabilitation in home-based stroke patients. Li et al. conducted a randomized, double-blind, sham-controlled trial involving 30 subacute stroke patients (53). Over 3 weeks, participants received taVNS combined with task-oriented training. Results demonstrated that compared to the sham stimulation group, the taVNS group achieved significant improvements in cognitive function and limb motor function among patients with PSCI. Functional near-infrared spectroscopyc (fNIRS) data further demonstrated that taVNS activated the prefrontal cortex, DLPFC, and primary motor cortex—regions associated with cognition and motor function. This study indicates that taVNS promotes post-stroke recovery of cognitive and upper limb function through multi-level mechanisms by activating cognition-motor-related brain regions and facilitating network functional reorganization. It provides novel neurobiological insights and intervention pathways for stroke rehabilitation.

### 4.3 Application of taVNS in chronic kidney disease-related cognitive impairment

Maintenance hemodialysis (MHD) is the primary renal replacement therapy for end-stage CKD (66). In recent years, the concept of the “kidney-brain axis” has gained increasing attention (67). Research indicates that hemodynamic fluctuations and osmotic pressure changes during hemodialysis (HD) may impose additional stress on the brain, potentially inducing ischemic injury and neurovascular damage, ultimately leading to HD-associated CI (68, 69). Reports indicate that the incidence of CI in MHD patients reaches 50 to 70%, and it has been identified as an independent risk factor for mortality (54, 70). Further studies using magnetic resonance imaging and spectroscopy during dialysis have confirmed the association between acute brain injury associated with maintenance hemodialysis and cognitive decline (71). Therefore, early intervention and treatment are crucial for improving patient prognosis.

Wang et al. conducted a randomized, single-blind, sham-stimulation-controlled trial involving 36 MHD patients to investigate taVNS intervention (54). Results demonstrated that compared with the sham-stimulation group, taVNS significantly improved cognitive function and reduced fatigue during dialysis. fNIRS monitoring revealed that taVNS effectively mitigated dialysis-induced decreases in DLPFC cerebral oxygen saturation, with significant correlations between cerebral oxygenation changes and cognitive performance. This study suggests that taVNS may alleviate cognitive decline in MHD patients by improving cerebral hypoxia during dialysis, offering a potential non-pharmacological intervention strategy for preventing and treating dialysis-related brain injury.

## 5 The underlying mechanism of taVNS in cognitive impairment

**Figure 4:**
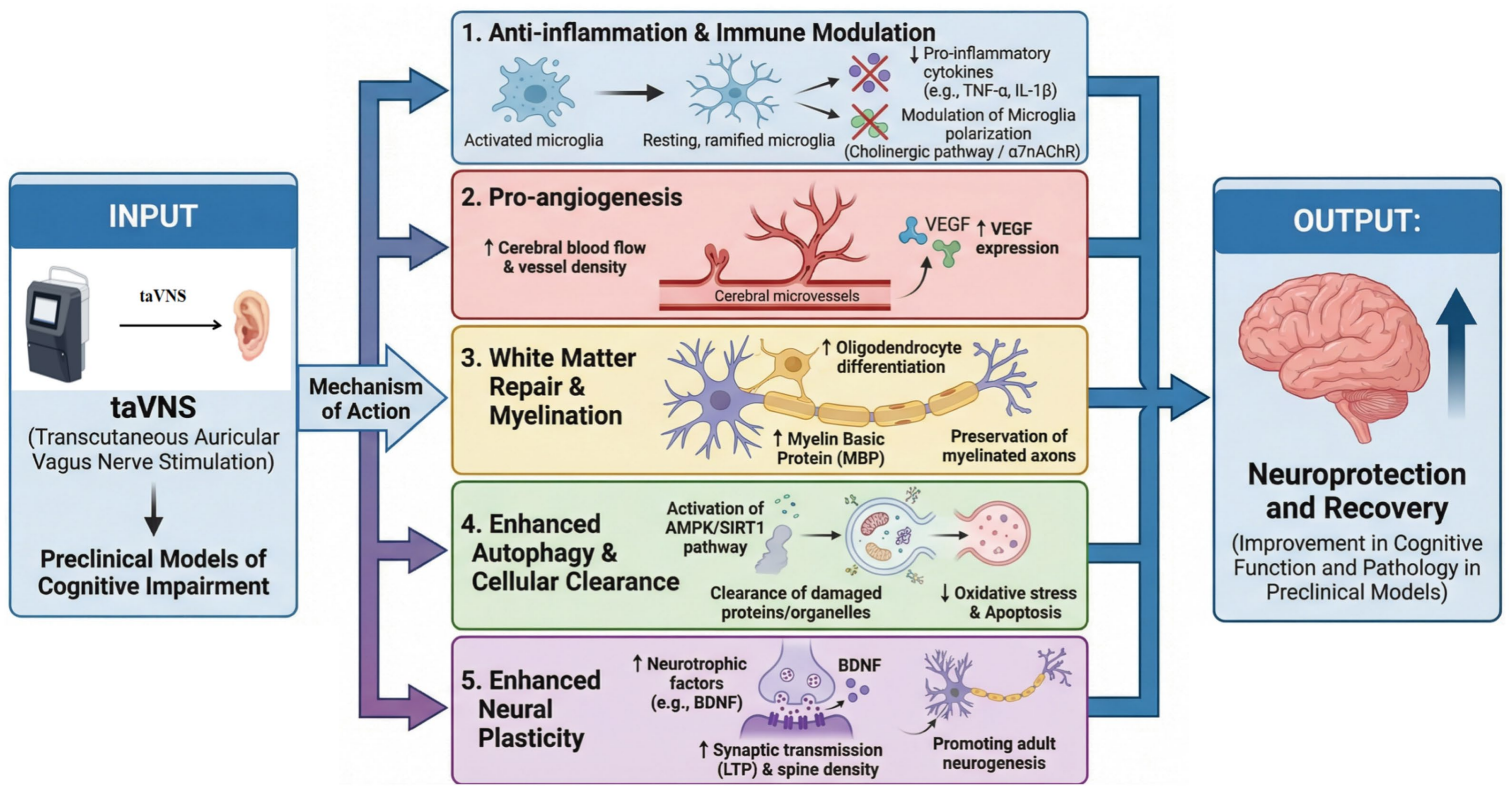
In preclinical models of cognitive impairment, taVNS exerts neuroprotective effects through five core pathways: (1) Anti-inflammation via cholinergic/α7nAChR-mediated microglial modulation, inhibiting cytokines; (2) Pro-angiogenesis through VEGF upregulation, enhancing cerebral perfusion; (3) White matter repair by promoting oligodendrocyte differentiation and MBP expression; (4) Enhanced autophagy via AMPK/SIRT1 activation, reducing oxidative stress and apoptosis; and (5) Enhanced neural plasticity through upregulated neurotrophic factors, improving LTP, dendritic spine density, and neurogenesis.

While clinical studies have confirmed the therapeutic potential of taVNS, the precise cellular and molecular mechanisms driving these improvements remain difficult to fully explore in human subjects due to ethical and technical constraints. To address this gap, this section synthesizes evidence from animal models to provide a biological explanation for the clinical benefits observed in Section 4. As illustrated in Figure 4 and Table 2, taVNS exerts multidimensional neuroprotective and restorative effects across various cognitive impairment models. Its effects are not dependent on a single pathway but are achieved through five interconnected core mechanisms thoroughly validated by preclinical research, presented here in the order they appear in the figure: anti-inflammation and immune modulation, pro-angiogenesis, white matter repair and myelination, enhanced autophagy and cellular clearance, and enhanced neural plasticity. These mechanisms precisely target key pathophysiological links in CI. Through synergistic effects, they improve the brain microenvironment, protect neuronal function, and ultimately promote cognitive recovery. The following sections detail the specific actions and molecular basis of each mechanism sequentially, supported by preclinical evidence from diverse cognitive impairment models.

**Table 2:** The underlying mechanism of taVNS in cognitive impairment.

References	Rodent models	Device	Initial time	taVNS parameter	Stimulation site	Key Biomarkers/Effects	Results and conclusion
Cai et al. (75)	Male Sprague–Dawley rats	taVNS	5 min before surgery	Frequency: 20 Hz Intensity: 0.5 mA Pulse width: 0.5 ms Stimulation duration: 30-s train repeated 4 times with 5-min intervals	Left cavum concha	α7nAChR expression; Pro-inflammatory cytokines; NF-κB activation; Apoptosis markers; AD-related pathology	1. Improved spatial memory 2. Reduced neuroinflammation 3. Decreased hippocampal apoptosis 4. Attenuated AD-related pathology
Choi et al. (89)	Male C57BL/6 mice with tBCCAO-induced VCI	taVNS	Post-surgery; Behavioral tests on day 3 (short-term) or day 7 (long-term)	Frequency: 20 Hz Intensity: 1 mA Pulse width: 330 μs Duration: 20 min/session	Cymba concha and dorsal auricle of left ear	CSF circulation; Heart rate; Cognitive behavior	Enhanced CSF circulation Restored cognitive function (NOR) Effective in both short- and long-term regimens
Brambilla-Pisoni et al. (84)	Adult male CD-1 mice	atVNS	Immediately or 3 h after NOR training	Frequency: 20 Hz; Intensity: 1 mA; Pulse width: 320 μs; Duration: 30 min	Left auricular concha	c-Fos-based functional connectivity network; Novel object recognition memory performance	1. Enhanced memory persistence with critical time window 2. Unaltered regional neuronal activation in 30 brain regions 3. Reorganized functional brain network
Zhou et al. (76)	Aged male Sprague–Dawley rats	taVNS	24 h before anesthesia, for 5 days	Frequency: 10 Hz Intensity: 1 mA Pulse width: Not specified Stimulation duration: 30 min/day for 5 consecutive days	Left concha auricularis	Apoptosis; Necroptosis; Microglial activation; Cognitive function	Improved learning and memory (MWM) Activated cholinergic neurons; Reduced apoptosis and necroptosis Suppressed microglial activation and neuroinflammation Effects blocked by cholinergic lesion
Nazari et al. (90)	Male Wistar rats with morphine dependence and withdrawal	taVNS	Post-cessation, for 14 days	Frequency: 5, 20, 100 Hz Intensity: 2 mA Pulse Width: 500 μs Pattern: 30s ON, 5 min OFF Total Duration: 30 min/day	Left auricular concha region	Working/recognition memory; Anxiety-like behavior; Hippocampal mRNA	Ameliorated memory deficits Exerted anxiolytic effect Counteracted BDNF decrease and GFAP increase
Wang et al. (91)	Male BALB/c mice with acute stress	taVNS	1 day after stress induction	Frequency: 2, 5, 15, 25 Hz; 5 Hz was optimal Intensity: 1 mA Pulse Width: 200 μs Pattern: 120 s ON, 120 s OFF for 16 min total	Not specified	Hippocampal O-GlcNAc; IL-6/STAT3 pathway; Neuroinflammation; Cognitive behavior	Reversed memory deficits (NOR) Reduced O-GlcNAc levels and suppressed IL-6/STAT3 pathway Effect dependent on O-GlcNAc cycling
Gong et al. (87)	Adult male C57BL/6 J mice	atVNS	Day 3 post-stroke (subacute phase)	Frequency: 20 Hz; Intensity: 1 mA Pulse width: 330 ms Duration: 30 min/day for 5 consecutive days	Left auricular concha	Ferroptosis-related proteins Oxidative stress; Neurogenesis; Angiogenesis; Neuroinflammation	1. Inhibited ferroptosis reduced oxidative stress, and preserved mitochondrial integrity 2. Suppressed neuroinflammation 3. Enhanced neurogenesis and angiogenesis, leading to improved sensorimotor and cognitive function
Sun et al. (56)	Adult male Sprague–Dawley rats with tBCCAO-induced VCID	taVNS	Starting on post-surgery day 13, for 14 consecutive days	Frequency: 20 Hz; Duration: 30 min/day	Left auricular concha	Wnt7a, Wnt7b, β-catenin, p-GSK3β, VEGF, TJ proteins, FGL2 leakage, CD34 + microvessel density, GFAP, C3, S100A10, neuronal damage, apoptosis	1. Improved cognitive function 2. Reduced neuronal damage and apoptosis 3. Decreased BBB permeability and enhanced angiogenesis 4. Upregulated Wnt7/β-catenin pathway and its downstream targets

Alzheimer’s disease, AD; Morris Water Maze, MWM; cerebrospinal fluid, CSF; Novel Object Recognition, NOR; brain-derived neurotrophic factor, BDNF; glial fibrillary acidic protein, GFAP; Open Field Test, OFT.

### 5.1 Anti-inflammation and immune modulation

Anesthesia, surgical trauma, and ischemic events act as profound physiological stressors that can trigger widespread neuroinflammation, a pathological cascade that severely disrupts the neuronal microenvironment, impairs synaptic plasticity, and precipitates cognitive decline (72). This neuroinflammatory response is frequently initiated by the systemic release of damage-associated molecular patterns (DAMPs), which compromise the blood–brain barrier and provoke the rapid activation of microglia, the resident macrophages of the central nervous system (73). Upon activation, microglia typically undergo a phenotypic shift toward a neurotoxic, pro-inflammatory state, resulting in the excessive synthesis and release of detrimental cytokines, such as tumor necrosis factor-alpha (TNF-*α*), interleukin-1 beta (IL-1β), and interleukin-6 (IL-6) (74). taVNS exerts robust neuroprotective effects by actively modulating this microglial polarization. Rather than merely suppressing overall immune function, taVNS effectively shifts microglia from an activated, pro-inflammatory state back to a resting, ramified state, thereby promoting a neuroprotective microenvironment (75).

This sophisticated immune modulation is primarily mediated through the engagement of the cholinergic anti-inflammatory pathway (CAP). Mechanistically, ascending signals from taVNS activate the basal forebrain-hippocampal cholinergic system, prompting the localized release of acetylcholine (ACh). This ACh interacts with α7 nicotinic acetylcholine receptors (α7nAChR) densely expressed on microglial surfaces, triggering an intracellular signaling cascade that inhibits the nuclear translocation of nuclear factor-kappa B (NF-κB) and significantly downregulates the transcription of pro-inflammatory cytokines (75, 76). The indispensable role of this specific neural circuit has been rigorously validated: selective lesioning of basal forebrain cholinergic neurons with the immunotoxin 192 IgG-saporin (192-sap) completely abolishes the anti-inflammatory effects of taVNS. Ultimately, by alleviating cytokine-mediated neurotoxicity and resolving neuroinflammation through this target pathway, taVNS plays a vital role in restoring circuit homeostasis and protecting against post-operative or post-ischemic neurological deficits.

### 5.2 Promote angiogenesis

Beyond mitigating inflammation, facilitating the structural and functional regeneration of the neurovascular unit (NVU) is critical for sustained cognitive and neurological recovery (77). Following ischemic events, surgical trauma, or other forms of severe neurological injury, the central nervous system experiences a profound energetic crisis exacerbated by vascular damage (78). Specifically, the blood–brain barrier (BBB) often suffers severe structural disruption, leading to increased permeability, microvascular leakage, and significantly compromised local cerebral perfusion (79). This disruption creates a hostile, hypoxic microenvironment where neurons are deprived of essential oxygen and metabolic substrates, ultimately accelerating secondary brain injury and cognitive decline. Therefore, effective therapeutic strategies must extend beyond cellular neuroprotection to actively promote neurorestoration, with the reconstruction of a robust and stable microvascular network being paramount.

taVNS addresses this critical deficit by actively promoting structural remodeling and stimulating targeted angiogenesis to restore the damaged vascular network. Recent empirical evidence demonstrates that in models of vascular cognitive impairment, taVNS significantly attenuates BBB damage and accelerates the generation of new cerebral microvessels. At the molecular level, this neurovascular repair is driven by the marked upregulation of vascular endothelial growth factor (VEGF) expression and the subsequent activation of the canonical Wnt7/*β*-catenin signaling pathway (56). By engaging these critical morphogenic pathways, taVNS effectively increases regional cerebral blood flow and restores vessel density in the ischemic or injured penumbra. Ultimately, this robust pro-angiogenic effect provides the essential metabolic support and stable microenvironmental foundation required for long-term neuronal survival, synaptic plasticity, and comprehensive functional recovery.

### 5.3 White matter repair and myelination

The preservation of efficient neural communication networks relies heavily on the microstructural integrity of white matter tracts. Pathological processes underlying severe neurological injury and cognitive impairment—such as chronic ischemia, neuroinflammation, and metabolic stress—often disproportionately affect the highly vulnerable myelin sheaths and their myelinating cells, the mature oligodendrocytes (80, 81). This structural degradation is characterized by widespread demyelination and severe damage to myelinated axons, which directly abolishes saltatory conduction. Consequently, the loss of myelin integrity leads to severe signal attenuation, action potential failure, and the ultimate desynchronization of large-scale brain network activity, serving as a primary anatomical substrate for neurocognitive deficits (82).

taVNS intervenes decisively in this structural degradation by promoting robust white matter repair and remyelination (52). Mechanistically, the optimized neuroprotective microenvironment fostered by taVNS facilitates the recruitment, proliferation, and subsequent differentiation of local oligodendrocyte precursor cells (OPCs) into mature, functionally myelinating oligodendrocytes (83). This targeted cellular maturation leads to a significant upregulation in the synthesis and expression of Myelin Basic Protein (MBP), a crucial structural protein required for the compaction and stabilization of the newly formed myelin sheath. By actively enhancing this endogenous remyelination process and preserving the integrity of myelinated axons, taVNS successfully restores the critical physical foundation necessary for high-speed neural signal conduction, thereby rescuing structural connectivity and synchronized brain network dynamics (84).

### 5.4 Enhanced autophagy and cellular clearance

The progressive accumulation of damaged proteins, dysfunctional organelles, and severe oxidative stress inevitably triggers multiple programmed cell death pathways, leading to irreversible neuronal loss. This severe structural degradation serves as the primary pathological foundation for cognitive decline across various neurological disorders (54, 85). taVNS robustly counters this damage by enhancing multi-level cellular clearance and survival mechanisms. At the intracellular level, taVNS activates key metabolic sensors such as the AMPK/SIRT1 signaling pathway, which subsequently upregulates cellular autophagy to accelerate the degradation and clearance of toxic intracellular aggregates (86). By restoring intrinsic cellular homeostasis, taVNS significantly attenuates oxidative stress and potently inhibits mitochondrial apoptosis pathways (87). This anti-apoptotic effect is clearly evidenced by the profound downregulation of pro-apoptotic proteins, including Bax and cleaved caspase-3, alongside a concurrent increase in the protective Bcl-2/Bax ratio (75). Furthermore, expanding beyond classical apoptosis, recent evidence demonstrates that taVNS-mediated α7nAChR signaling effectively suppresses ferroptosis, underscoring its sophisticated, multi-target potential in comprehensive cellular protection (87).

Beyond these intracellular defense mechanisms, the preservation of a healthy neural microenvironment heavily depends on the efficient macroscopic clearance of extracellular metabolic waste. Under pathological conditions, the impairment of cerebrospinal fluid (CSF) dynamics leads to the toxic accumulation of neurodegenerative byproducts, further exacerbating secondary neuronal injury (88). Remarkably, taVNS addresses this systemic deficit by significantly enhancing CSF circulation through the brain’s glymphatic system. *In vivo* evidence demonstrates that taVNS interventions markedly accelerate the paracellular flow within perivascular spaces, facilitating the rapid and efficient clearance of critical neurotoxic macromolecules, such as amyloid-beta (89). By synergistically linking intracellular autophagic restoration with macroscopic glymphatic waste removal, taVNS comprehensively purifies the neural microenvironment, thereby establishing a robust biological foundation for halting disease progression and facilitating sustained cognitive recovery.

### 5.5 Enhanced neural plasticity

The ultimate recovery of cognitive function relies on reconstructing neural circuits through enhanced plasticity and neurogenesis. Under pathological conditions, disruptions in neurotransmitter equilibrium lead to impaired long-term potentiation (LTP) and synaptic structural damage (76, 90). taVNS addresses this by significantly upregulating the expression of key neurotrophic factors, particularly brain-derived neurotrophic factor (BDNF). This upregulation enhances synaptic transmission efficiency (LTP) and increases dendritic spine density, thereby directly stabilizing synaptic plasticity. Simultaneously, taVNS actively promotes adult neurogenesis. By coupling with angiogenesis to create a favorable “neurovascular niche,” taVNS facilitates the proliferation of new neurons and their integration into existing neural networks (87). At the macro-network level, this enhanced plasticity reorganizes whole-brain functional connectivity (84) and restores synaptic modifications (91), driving robust recovery in learning, memory, and information processing.

## 6 Current challenges and future directions

### 6.1 Lack of standardized treatment protocols and parameter consensus

Currently, the core bottleneck in taVNS clinical research and application lies in the lack of unified, standardized treatment protocols. This deficiency is most evident in the significant heterogeneity of key stimulation parameters. Regarding core parameters, frequencies range from 4 Hz to 100 Hz, pulse widths vary between 200 and 500 microseconds, and intensities fluctuate between 0.5 and 10 milliamps; Treatment durations also vary considerably, ranging from as short as 2 weeks to as long as 24 weeks. This ambiguity in treatment “dosage” severely hinders the reproducibility and comparability of research findings, introducing uncertainty for clinical implementation. The root cause lies in the fact that most parameter selections still rely on empirical guidance, lacking clear theoretical foundations grounded in neural mechanisms. Consequently, the optimal stimulation protocol remains shrouded in a “black box,” posing a critical obstacle to the field’s advancement.

The underlying reasons for such parameter variability are multifaceted. First, differences in device manufacturers and models lead to inherent variations in output characteristics, waveform profiles, and impedance-matching capabilities (92). Second, individual anatomical differences—such as auricular morphology, skin thickness, and subcutaneous tissue composition—affect local impedance and current distribution, thereby influencing the effective dose delivered to the vagal afferents (37). Third, the choice of stimulation site not only determines the density of vagal innervation but also alters the perceived sensation and potential engagement of non-vagal fibers, which may further complicate parameter translation across studies (96). Another critical methodological issue concerns the design of control or sham interventions. Commonly used sham approaches include stimulation of non-vagal innervated areas or the use of devices with identical appearance but sub-threshold or no current output. However, these methods may not fully control for placebo effects, somatosensory confounding, or participant expectation, because different ear locations exhibit distinct cutaneous innervation and perceptual qualities. Moreover, inadequate blinding due to perceptible differences between active and sham stimulation can bias outcome assessments, particularly in subjective cognitive measures (95). To date, no universally accepted sham protocol has been established, and existing guidelines—such as those proposed by the International Federation of Clinical Neurophysiology or consensus statements on non-invasive vagus nerve stimulation—remain preliminary and lack enforcement in trial design (49, 97).

To advance taVNS from a promising technology into a truly reliable therapy, future efforts must focus on establishing a systematic standard framework. The primary task is to adopt a “mechanism-guided parameter optimization” research paradigm. Preclinical studies should clarify the differential regulatory effects of different parameters on specific neural pathways—including the cholinergic system and neuroinflammatory responses—thereby providing a robust biological foundation for clinical protocol design. Simultaneously, the field urgently requires the formulation and implementation of mandatory clinical research reporting standards to ensure comprehensive disclosure and transparency of all core stimulation parameters and device information. Long-term efforts should actively explore the use of neuroimaging and physiological biomarkers to advance treatment strategies toward individualized, precision medicine approaches. Ultimately, this will elevate taVNS into a standardized intervention characterized by clear efficacy, well-defined protocols, and high reproducibility.

### 6.2 Challenges of limited sample size and placebo effects

It is important to note that while a broad literature search yields numerous records related to VNS and cognition, the number of studies meeting the specific criteria for clinical taVNS interventions in organic cognitive impairment remains limited, reflecting the emerging nature of this field. Currently, clinical research on taVNS in the field of CI remains in its preliminary exploratory phase, with a common issue being limited sample sizes. As shown in Table 1, most trials involve only dozens of participants, and some studies are even case reports. Small-sample studies have low statistical power, making it difficult to identify clinically meaningful subtle cognitive improvements while also limiting the generalizability and stability of research findings. Furthermore, the interference of placebo effects cannot be overlooked. Although some studies have attempted to use non-Vagus nerve distribution areas such as the tragus or earlobe as sham stimulation controls, methodological controversy persists regarding whether such control methods can fully replicate the sensory experience of genuine stimulation while avoiding neuromodulatory effects. If placebo controls are not rigorously designed, it becomes challenging to distinguish the specific therapeutic effects of taVNS from non-specific effects driven by participants’ psychological expectations. Therefore, large-scale, multicenter randomized controlled trials with rigorously designed sham-stimulation controls are urgently needed to validate the therapeutic specificity of taVNS. Concurrently, particular attention should be paid to tolerability in special populations to provide more robust evidence-based medical support for the clinical translation of taVNS.

### 6.3 Insufficient depth in mechanism research

Current studies on the mechanisms of taVNS predominantly focus on a limited number of molecules or pathways, such as BDNF, NF-κB, and acetylcholine. While these studies have preliminarily identified potential intervention targets, the research perspective remains relatively narrow. There is a lack of multi-omics integration at the systems level, making it difficult to comprehensively characterize the overall network through which taVNS regulates neural function. Existing mechanism explorations largely rely on traditional molecular biology methods, which remain incapable of systematically deciphering complex processes such as cell type specificity, spatiotemporal dynamics, and multi-pathway synergistic mechanisms involved in neural regulation. Although systems biology approaches like single-cell sequencing, spatial transcriptomics, and proteomics have demonstrated potential for genome- or proteome-wide mechanistic analysis (61, 93), their application in taVNS research remains insufficient.

Therefore, there is an urgent need to introduce systems biology research strategies that integrate multi-level information encompassing molecular interactions, cellular communication, neural circuits, and behavioral phenotypes to construct a comprehensive taVNS regulatory network. Integrative analysis at the systems level will facilitate the accurate identification of key regulatory nodes, the discovery of novel therapeutic targets, and provide robust experimental evidence for the clinical application of taVNS.

### 6.4 Artificial intelligence and personalized treatment urgently require development

The rapid advancement of artificial intelligence (AI) offers new opportunities for optimizing taVNS treatment strategies. Future research should systematically integrate multimodal data to build AI-based systems for individualized efficacy prediction and parameter optimization. Specifically, machine learning and deep learning algorithms can be harnessed in the following ways to advance personalized taVNS therapy: First, AI can be used to identify taVNS-responsive biotypes by analyzing neuroimaging data such as fMRI, fNIRS, and EEG. Unsupervised clustering or feature-extraction methods could delineate distinct neural subtypes based on resting-state or task-based functional connectivity patterns—for example, within the default mode, salience, or prefrontal-limbic networks—and predict which patients are most likely to show cognitive improvement with taVNS. Second, real-time neurofeedback technologies could support the development of closed-loop taVNS systems that dynamically adjust stimulation frequency, intensity, or timing based on instantaneous neural responses, such as theta/alpha power or prefrontal oxygenation, enabling adaptive neuromodulation. Third, multimodal prediction models that integrate clinical, genomic, proteomic, and neuroimaging features—for instance, using graph neural networks or ensemble learning—could identify key biomarker combinations that influence taVNS efficacy, allowing pre-treatment outcome forecasting.

Together, these AI-driven strategies may help transition taVNS from an experience-guided, one-size-fits-all intervention toward a data-driven era of precision neuromodulation. User-friendly AI-assisted decision tools could eventually support clinicians in prescribing “personalized taVNS recipes”—including optimal stimulation sites, parameters, and treatment duration—while federated learning across multicenter datasets would enhance model generalizability and clinical applicability.

### 6.5 Collaborative applications require further research

TaVNS, as a neurostimulation technique with research value, demonstrates potential for synergistic application with various cognitive impairment interventions. Existing explorations involve combining taVNS with cognitive training, pharmacotherapy, and other neurostimulation techniques. Preliminary studies suggest possible additive effects in improving working memory and executive function, though further evidence is needed. Future research should focus on determining optimal sequencing and parameter configurations for different intervention combinations, gradually establishing personalized combination treatment protocols. This approach will advance cognitive impairment intervention strategies from “single-modality” to more precise “combination interventions”.

While this review primarily focuses on cognitive impairment stemming from neurological and organic pathologies, cognitive deficits in patients with major depressive disorder—often manifesting as “depressive pseudodementia”—are garnering increasing scholarly attention. The pathogenesis of depression-related cognitive impairment is closely linked to emotional dysregulation, neuroinflammation, and neurotransmitter imbalances. Notably, these pathological factors share significant overlap with the potential therapeutic mechanisms of taVNS discussed herein, particularly its anti-inflammatory and autonomic regulatory effects. Consequently, future research should extend to evaluating the therapeutic efficacy of taVNS in depression-associated cognitive impairment and further clarify the specific neurobiological mechanisms underlying its effects in this distinct population. Additionally, future studies should also consider the preventive potential of taVNS in healthy aging populations, as reviewed by Naparstek et al. (94), which may offer a novel strategy for delaying the onset of neurodegenerative conditions.

## 7 Limitations of this review

First, the types of cognitive impairment included in the studies exhibited high heterogeneity, encompassing MCI, PSCI and chronic kidney disease-related CI. While this reflects the breadth of the taVNS research field, the fundamentally different pathophysiological mechanisms underlying these distinct cognitive impairment subtypes may result in markedly divergent response patterns to interventions. This poses methodological constraints on cross-comparison of study results and interpretation of underlying mechanisms. Second, the current number of high-quality clinical studies targeting specific cognitive impairment subtypes is limited, and significant clinical heterogeneity exists in terms of subject inclusion criteria, stimulation parameter protocols, and efficacy assessment metrics. Consequently, this review could not perform a quantitative meta-analysis and primarily employed qualitative synthesis and descriptive analysis methods. This methodological limitation not only affects the accurate assessment of the overall effect size of taVNS but also constrains the feasibility of exploring potential influencing factors through subgroup analysis. Third, in clinical practice, taVNS is often combined with other interventions such as drug therapy or rehabilitation training. However, this review was unable to systematically evaluate the potential interaction effects between taVNS and these interventions. This limitation impacts the generalizability of the findings to real-world clinical settings. Additionally, the inclusion of different study designs, ranging from RCTs to case reports, introduces variability in the strength of evidence. While case reports were included to provide mechanistic insights and feasibility data in this emerging field, their inherent lack of generalizability limits the strength of conclusions compared to large-scale randomized trials. Finally, existing literature on taVNS mechanisms primarily relies on correlational evidence from diverse research contexts, lacking direct experimental validation of specific causal pathways. This incomplete evidence chain limits the strength of current mechanistic hypotheses, whose reliability requires further validation through rigorously designed future mechanism studies.

## 8 Conclusion

In summary, current evidence suggests taVNS as a promising non-invasive neuromodulation approach for cognitive impairment. Preclinical studies demonstrate its multi-mechanistic neuroprotective effects, while preliminary clinical reports show potential benefits across various cognitive impairment types, including mild cognitive impairment, post-stroke cognitive impairment, and chronic kidney disease-related cognitive decline. However, the clinical evidence remains limited by small sample sizes, methodological heterogeneity, and insufficient long-term data. Significant challenges persist in parameter standardization, sham control design, and mechanistic understanding. While taVNS represents an accessible therapeutic avenue worthy of continued exploration, its clinical efficacy and optimal application parameters require validation through larger, well-controlled trials. Future research should focus on establishing standardized protocols and elucidating response mechanisms to determine taVNS’s potential role in cognitive impairment management.
